# Supplementary material for: The effect of physical exercise on depression among college students: a systematic review and meta-analysis
Source: PeerJ. 2024 Sep 23;12:e18111. doi: 10.7717/peerj.18111 (PMC11426321; doi:10.7717/peerj.18111)
Supplement: Supplemental Information 1 [file peerj-12-18111-s001.docx]

Records identified through

database searching (n=203)

PubMed(n=30)

Embase(n=113)

Cochrane library(n=10)

Web of science(n=50)

··

Records removed before screening:

Duplicate records removed (n =26)

**Identification**

Records excluded after reading the title and abstract

(n =146)

Records after removal of

duplicates(n=177)

Exclude records(n=21)

-The full text is not

available(n=10)

-The study population does not meet (n=6)

-No available data(n=4)

-No control group(n=3)

Full-text articles evaluated

for eligibility (n=31)

**Screening**

Reports assessed for eligibility (n =8)

Studies included in quantitative synthesis (Meta-analysis)

(n =8)

**Included**
