# Supplementary material for: The effect of physical exercise on depression among college students: a systematic review and meta-analysis
Source: PeerJ. 2024 Sep 23;12:e18111. doi: 10.7717/peerj.18111 (PMC11426321; doi:10.7717/peerj.18111)
Supplement: Supplemental Information 3 [file peerj-12-18111-s003.docx]

## Appendix A

Table A1. Database-specific search strategies using the PubMed database as an example

| Database | Search Strategy |
| --- | --- |
| PubMed | ("Depression"[MeSH Terms] OR ("Depression"[MeSH Terms] OR "Depression"[All Fields] OR ("depressive"[All Fields] AND "symptoms"[All Fields]) OR "depressive symptoms"[All Fields] OR ("Depression"[MeSH Terms] OR "Depression"[All Fields] OR ("depressive"[All Fields] AND "symptom"[All Fields]) OR "depressive symptom"[All Fields]) OR ("Depression"[MeSH Terms] OR "Depression"[All Fields] OR ("symptom"[All Fields] AND "depressive"[All Fields]) OR "symptom depressive"[All Fields]) OR ("Depression"[MeSH Terms] OR "Depression"[All Fields] OR ("emotional"[All Fields] AND "Depression"[All Fields]) OR "emotional depression"[All Fields]) OR ("Depression"[MeSH Terms] OR "Depression"[All Fields] OR ("Depression"[All Fields] AND "emotional"[All Fields]) OR "depression emotional"[All Fields]))) AND ("Exercise"[MeSH Terms] OR ("Exercise"[MeSH Terms] OR "Exercise"[All Fields] OR "exercises"[All Fields] OR "exercise therapy"[MeSH Terms] OR ("Exercise"[All Fields] AND "therapy"[All Fields]) OR "exercise therapy"[All Fields] OR "exercising"[All Fields] OR "exercise s"[All Fields] OR "exercised"[All Fields] OR "exerciser"[All Fields] OR "exercisers"[All Fields] OR ("Exercise"[MeSH Terms] OR "Exercise"[All Fields] OR ("physical"[All Fields] AND "activity"[All Fields]) OR "physical activity"[All Fields]) OR ("Exercise"[MeSH Terms] OR "Exercise"[All Fields] OR ("activities"[All Fields] AND "physical"[All Fields]) OR "activities physical"[All Fields]) OR ("Exercise"[MeSH Terms] OR "Exercise"[All Fields] OR ("activity"[All Fields] AND "physical"[All Fields]) OR "activity physical"[All Fields]) OR ("Exercise"[MeSH Terms] OR "Exercise"[All Fields] OR ("physical"[All Fields] AND "activities"[All Fields]) OR "physical activities"[All Fields]) OR ("Exercise"[MeSH Terms] OR "Exercise"[All Fields] OR ("Exercise"[All Fields] AND "physical"[All Fields]) OR "exercise physical"[All Fields]) OR ("Exercise"[MeSH Terms] OR "Exercise"[All Fields] OR ("exercises"[All Fields] AND "physical"[All Fields]) OR "exercises physical"[All Fields]) OR ("Exercise"[MeSH Terms] OR "Exercise"[All Fields] OR ("physical"[All Fields] AND "Exercise"[All Fields]) OR "physical exercise"[All Fields]) OR ("Exercise"[MeSH Terms] OR "Exercise"[All Fields] OR ("physical"[All Fields] AND "exercises"[All Fields]) OR "physical exercises"[All Fields]) OR ("Exercise"[MeSH Terms] OR "Exercise"[All Fields] OR ("acute"[All Fields] AND "Exercise"[All Fields]) OR "acute exercise"[All Fields]) OR ("Exercise"[MeSH Terms] OR "Exercise"[All Fields] OR ("acute"[All Fields] AND "exercises"[All Fields]) OR "acute exercises"[All Fields]) OR ("Exercise"[MeSH Terms] OR "Exercise"[All Fields] OR ("Exercise"[All Fields] AND "acute"[All Fields]) OR "exercise acute"[All Fields]) OR ("Exercise"[MeSH Terms] OR "Exercise"[All Fields] OR ("exercises"[All Fields] AND "acute"[All Fields]) OR "exercises acute"[All Fields]) OR ("Exercise"[MeSH Terms] OR "Exercise"[All Fields] OR ("Exercise"[All Fields] AND "isometric"[All Fields]) OR "exercise isometric"[All Fields]) OR ("Exercise"[MeSH Terms] OR "Exercise"[All Fields] OR ("exercises"[All Fields] AND "isometric"[All Fields]) OR "exercises isometric"[All Fields]) OR ("Exercise"[MeSH Terms] OR "Exercise"[All Fields] OR ("isometric"[All Fields] AND "exercises"[All Fields]) OR "isometric exercises"[All Fields]) OR ("Exercise"[MeSH Terms] OR "Exercise"[All Fields] OR ("isometric"[All Fields] AND "Exercise"[All Fields]) OR "isometric exercise"[All Fields]) OR ("Exercise"[MeSH Terms] OR "Exercise"[All Fields] OR ("Exercise"[All Fields] AND "aerobic"[All Fields]) OR "exercise aerobic"[All Fields]) OR ("Exercise"[MeSH Terms] OR "Exercise"[All Fields] OR ("aerobic"[All Fields] AND "Exercise"[All Fields]) OR "aerobic exercise"[All Fields]) OR ("Exercise"[MeSH Terms] OR "Exercise"[All Fields] OR ("aerobic"[All Fields] AND "exercises"[All Fields]) OR "aerobic exercises"[All Fields]) OR ("Exercise"[MeSH Terms] OR "Exercise"[All Fields] OR ("exercises"[All Fields] AND "aerobic"[All Fields]) OR "exercises aerobic"[All Fields]) OR ("Exercise"[MeSH Terms] OR "Exercise"[All Fields] OR ("Exercise"[All Fields] AND "training"[All Fields]) OR "exercise training"[All Fields]) OR ("Exercise"[MeSH Terms] OR "Exercise"[All Fields] OR ("Exercise"[All Fields] AND "trainings"[All Fields]) OR "exercise trainings"[All Fields]) OR ("Exercise"[MeSH Terms] OR "Exercise"[All Fields] OR ("training"[All Fields] AND "Exercise"[All Fields]) OR "training exercise"[All Fields]) OR ("Exercise"[MeSH Terms] OR "Exercise"[All Fields] OR ("trainings"[All Fields] AND "Exercise"[All Fields])))) AND ("randomized controlled trial"[Publication Type] OR "randomized controlled trials as topic"[MeSH Terms] OR "randomized controlled trials"[All Fields] OR "randomised controlled trials"[All Fields] OR ("randomized controlled trial"[Publication Type] OR "randomized controlled trials as topic"[MeSH Terms] OR "clinical trials randomized"[All Fields] OR "clinical trials randomised"[All Fields] OR ("randomized controlled trial"[Publication Type] OR "randomized controlled trials as topic"[MeSH Terms] OR "trials randomized clinical"[All Fields] OR "trials randomised clinical"[All Fields]) OR ("randomized controlled trial"[Publication Type] OR "randomized controlled trials as topic"[MeSH Terms] OR "controlled clinical trials randomized"[All Fields] OR "controlled clinical trials randomised"[All Fields]))) AND ("students, public health"[MeSH Terms] OR ("students, public health"[MeSH Terms] OR ("students"[All Fields] AND "public"[All Fields] AND "health"[All Fields]) OR "public health students"[All Fields] OR ("health"[All Fields] AND "student"[All Fields] AND "public"[All Fields]) OR ("students, public health"[MeSH Terms] OR ("students"[All Fields] AND "public"[All Fields] AND "health"[All Fields]) OR "public health students"[All Fields] OR ("health"[All Fields] AND "students"[All Fields] AND "public"[All Fields]) OR "health students public"[All Fields]) OR ("students, public health"[MeSH Terms] OR ("students"[All Fields] AND "public"[All Fields] AND "health"[All Fields]) OR "public health students"[All Fields] OR ("public"[All Fields] AND "health"[All Fields] AND "student"[All Fields]) OR "public health student"[All Fields]) OR ("students, public health"[MeSH Terms] OR ("students"[All Fields] AND "public"[All Fields] AND "health"[All Fields]) OR "public health students"[All Fields] OR ("student"[All Fields] AND "public"[All Fields] AND "health"[All Fields]) OR "student public health"[All Fields]) OR ("students, public health"[MeSH Terms] OR ("students"[All Fields] AND "public"[All Fields] AND "health"[All Fields]) OR "public health students"[All Fields] OR ("public"[All Fields] AND "health"[All Fields] AND "students"[All Fields])))) |
