# Supplementary material for: The effect of physical exercise on depression among college students: a systematic review and meta-analysis
Source: PeerJ. 2024 Sep 23;12:e18111. doi: 10.7717/peerj.18111 (PMC11426321; doi:10.7717/peerj.18111)
Supplement: Supplemental Information 4 [file peerj-12-18111-s004.docx]

## **Date Search Was Performed**

The date on which the search was conducted was indicated in the original submission of the manuscript, and the search at line 121 included databases such as PubMed, Embase, Cochrane Library and Web of Science, covering publications up to 21 February 2024.

## **Systematic Review and/or Meta-Analysis Rationale**

**For systematic reviews / meta-analyses, authors need to provide the following information:**

1. **The rationale for conducting the systematic review / meta-analysis;**

The prevalence of major depression reaches its highest point among 20-24 year olds, and again college students are within this age group.

The proportion of college students afflicted with psychological well-being problems including stress, anxiety, or depression has significantly increased in recent year.College students go through enormous life changes, including away from their families , acquiring the ability to live autonomously, meeting new friends, and adjusting to increased academic responsibilities.These difficulties often arise in correlation with an increase in heightened levels of stress, anxiety, and even depression among college students.

Compared to previous research, there have been fewer studies investigating physical exercise therapies for treating depression among college students compared to adults. Furthermore, the previous studies provided evidence of significant and varying effects of physical exercise on depression, nevertheless, there still needs to be more clarity regarding the optimal form, intensity, duration, and frequency of physical exercise

1. **The contribution that it makes to knowledge in light of previously published related reports, including other meta-analyses and systematic reviews.**

Firstly, after the results of this study, it was found that physical exercise interventions had a significant improvement on depression in college students, as in different populations of previous studies, and this study can be used as a guidance programme for college students' physical exercise to improve their physical and mental health.Furthermore,physical exercise can reduce depression and may be an adjunct to medication and/or alternative treatments. Considering the findings of this study, it is important that health professionals (e.g., exercise physiologists, physicians, nurses, psychologists) promote physical exercise as a complementary alternative and take early action to prevent the worsening of depression.
